# Desensitizing efficacy of a universal dentin adhesive containing mesoporous bioactive glass on dentin hypersensitivity: a randomized clinical trial with a split-mouth model

**DOI:** 10.1038/s41598-024-64404-x

**Published:** 2024-06-17

**Authors:** Hyun-Jung Kim, Soram Oh, Jiyoung Kwon, Kyoung-Kyu Choi, Ji-Hyun Jang, Duck-Su Kim

**Affiliations:** 1 Department of Conservative Dentistry, School of Dentistry, Kyung Hee University, 24 Kyungheedae-ro, Dongdaemoon-gu, Seoul, 02453 South Korea; 2 Department of Conservative Dentistry, Kyung Hee University Dental Hospital, 26 Kyungheedae-ro, Dongdaemun-gu, Seoul, 02447 South Korea

**Keywords:** Randomized clinical study, Split-mouth design, Dentin hypersensitivity, Universal adhesive, Mesoporous bioactive glass, Desensitizing agent, Dental biomaterials, Bonded restorations

## Abstract

This split-mouth blinded randomized controlled study compared the efficacy of a desensitizing agent with oxalate/resin polymer and a universal adhesive containing mesoporous bioactive glass (MBG) for dentin hypersensitivity (DH) relief, using Schiff sensitivity score (SSS) and visual analog scale (VAS). Split quadrants containing teeth with DH were treated with either MS Coat ONE or Hi-Bond Universal with MBG as the functional additive. Assessments at baseline, immediately post-application, and at 1- and 2-week follow-ups used standardized stimulus protocols (air, cold, and acid). The SSS difference was the primary outcome, while the VAS difference was the secondary outcome. A mixed linear effect model performed statistical analysis. Immediate DH reduction occurred in response to air stimuli, with a significant decrease in Group HB than in Group MS (*p* = 0.0178). Cold stimulus reduction exhibited a gradual cumulative effect, with consistently greater reductions in Group HB than in Group MS (*p* ≤ 0.0377). Both groups effectively managed acidic stimuli, with no significant differences (*p* > 0.05). The VAS scores decreased gradually over the follow-up period (*p* < 0.0001). This study highlights the differential efficacy of treatments for various DH triggers and recommends specific approaches based on different stimulus types. The universal adhesive containing MBG demonstrated DH relief potential, promising efficacy identical to or superior to that of a dedicated desensitizing agent. Further research exploring the long-term efficacy and underlying mechanisms is warranted. The universal adhesive containing MBG can be adopted as an in-office desensitizing agent for DH relief. The desensitizing efficacy of universal adhesive matches or surpasses dedicated agents for air and cold stimuli.

## Introduction

Dentin hypersensitivity (DH), which is characterized by sharp and transient pain arising from exposed dentin in response to external stimuli, remains a significant concern in dentistry^1^. This condition not only leads to discomfort or unpleasant sensations in patients but also poses challenges for dental practitioners in terms of diagnosis and treatment. The diagnosis of DH typically excludes other forms of dental diseases or pathology^2,3^. Practically, the most common trigger of DH is a cold stimulus. For instance, exposure to cold air when breathing through the mouth as well as contact with air from the air/water syringe of a dental chair may trigger hyperesthesia^4,5^. DH pain can also be triggered by chemical stimuli, such as acidic or sweet foods^2,6^. Although several hypotheses have been proposed to date^7,8^, Brännström’s hydrodynamic theory is the most accepted theory for elucidating DH^9,10^. The theory claims that a stimulus to an exposed dentin surface increases fluid flow in the tubules, which in turn causes pressure changes across the dentin, activating the pulp-dentin border within the dentin tubules^9,11^. Närhi et al. state that sensitive dentin is a result of stimulus-induced fluid flow in the dentinal tubules and consequent nociceptor activation in the pulp-dentin border area^12^. This study also demonstrated that the patency of dentinal tubules is an important characteristic of sensitive dentin, with a significantly positive correlation between tubule density and pain responses induced by the exposed cervical dentin surfaces^12^.

Surprisingly, multiple treatment modalities exist for managing DH. The most common form of management is the application of a topical agent either by a dental professional or by the patient at home^13^. Two major strategies for managing DH exist^4,14^. First, certain therapeutic agents diminish neural transmission. Potassium nitrate is the most commonly used therapeutic component for nerve desensitization^4^. Additionally, potassium nitrate is currently used in most over-the-counter desensitization toothpaste and home- and in-office desensitizing gels^15–17^. This mechanism is attributed to the potassium nitrate’s ability to increase the extracellular potassium ion concentration, consequently depolarizing the nerve and preventing it from re-polarizing^16^. Another approach involves occluding the already opened tubules or creating coagulates inside the tubules^14^. This second strategy ranges from the use of ions, salts, or proteins to plug tubules, to the application of dentin sealers (adhesives or restorative materials, e.g., glass ionomers and composite resins) designed for physical blocks^4,13,14^. Some ions and salts, such as strontium salts, fluoride, and oxalates, are used to manage DH by forming precipitates within the dentinal tubules, consequently blocking dentinal fluid flow^18^. Composite resins and adhesives have been recommended for managing DH^19^.

Bioactive glass has been developed since Dr. Larry L. Hench invented it in 1969^20^. Additionally, bioactive glass has various abilities, such as bone regeneration, treatment of DH, and potential scaffolding^21^. In the field of dentistry, bioactive glass has been incorporated into dentin adhesives to enhance the durability of dentin bonding^22^. In addition to improving the resin–dentin bond, bioactive glass was assumed to decrease DH via dentin remineralization^23^. Recently, mesoporous (2–50 nm in pore size) bioactive glass (MBG)-containing universal dentin adhesives was released to the market (Hi-Bond Universal; MEDICLUS, Cheong-Ju, Korea). MBG demonstrated higher bioactivity than that displayed by conventional sol–gel-derived bioactive glass^24^. According to the manufacturer’s instructions, this adhesive can alleviate DH just like conventional desensitizing agents. This clinical study was designed to evaluate the effectiveness of Hi-Bond Universal in alleviating DH. We selected a conventional desensitizing agent as the control agent. A direct comparison of their desensitizing efficacy is lacking in the current literature. Therefore, this randomized clinical trial aimed to bridge this gap by evaluating and comparing the effectiveness of a dedicated dentin desensitizing agent containing a resin component, with that of a universal dentin adhesive containing a mineralizing biomaterial. This study used a split-mouth model, allowing for intra-individual comparisons and minimizing the effect of confounding variables.

The objective of this prospective randomized clinical trial was to compare the DH-relieving efficacy of a desensitizing agent mainly containing oxalates and a resin polymer with that of a universal adhesive containing MBG as a functional component, against various stimuli (air, cold, or acid) using Schiff sensitivity score (SSS) and visual analog scale (VAS).

The null hypotheses tested were the following:

1. The efficacy of relieving DH in response to air, cold, and acidic stimuli does not differ significantly according to the type of agent or follow-up period.
2. Subjective VAS scores for DH do not differ significantly during the follow-up period.

## Methods

### Study design

This was a prospective double-blind randomized controlled trial with a split-mouth design, involving patients who visited the Department of Conservative Dentistry of Kyung Hee University Dental Hospital. Ethical approval was obtained from the Institutional Review Board (IRB D22-001–001) of the Kyung Hee University Dental Hospital, and the study was registered with the Clinical Research Information Service (KCT0008101). To preserve the integrity of the blinding process in the single-site split-mouth clinical trial, our research team was strategically divided into two distinct units: an unblinded team and a blinded team. The unblinded team was responsible for the application of the DH agents, ensuring proper and consistent implementation of the clinical phase of our study. Concurrently, to maintain the objectivity of our clinical evaluations, the blinded team was tasked exclusively with the assessment of clinical outcomes (SSS) without any knowledge of the treatment that was applied.

To bolster our study’s robustness against potential biases, a dedicated researcher—who was not involved in the clinical procedures—was appointed to manage the data from both the unblinded and blinded teams. This researcher was solely responsible for the generation and handling of random codes, which were crucial for allocating treatments in a manner congruent with our split-mouth design. These measures ensured a strict separation of duties, thereby increasing the credibility of our findings by minimizing the risk of bias due to prior knowledge of treatment allocation.

Written informed consent was obtained from all the participants. The Consolidated Standards of Reporting Trials guidelines were adhered to. This clinical trial was conducted in accordance with the Ethics Code of the World Medical Association (Declaration of Helsinki). The study was conducted between November 2022 and June 2023.

### Sample size determination

The sample size was determined based on previous trials using G Power^25,26^. Sample size calculations were performed for two-paired means with a 1:1 allocation ratio, assuming normality, in a two-sided test with a split-mouth design. The present study was a paired observational study, as both the intervention and comparison treatments were applied to the same patient. This design is efficient as the sites that received interventions were similar, thus reducing variance and sample size requirements. In the present study, detecting a clinically significant difference (0.50 units following treatment as measured on the SSS, with a standard deviation of 1.00 units [effective size, f = 0.5]) required a sample size of 34 participants for the two treatment methods. Considering a dropout rate of 10–20%, 40 participants were enrolled.

### Inclusion and exclusion criteria

#### Inclusion

1. Participants aged 20 years or above.
2. Participants who were able to provide written informed consent.
3. Participants who were confirmed as healthy by the examiner, without clinically significant diseases that can interfere with the study outcomes.
4. Participants with more than two sensitive teeth with dentin exposure due to gingival recession, abrasion, and erosion

#### Exclusion

1. Individuals who received treatment for DH within the past 4 weeks
2. Individuals who had teeth with DH due to other reasons (e.g., dental caries and defective restorations)
3. Individuals who had taken nonsteroidal anti-inflammatory drugs or narcotic analgesics within the past week
4. Individuals with clinically significant allergic diseases
5. Pregnant or lactating women
6. Smokers
7. Individuals who used toothpaste specifically designed for treating DH in the past 4 weeks
8. Individuals who may have undergone teeth bleaching treatment within the past 4 weeks
9. Individuals who underwent scaling or periodontal treatment within the past 4 weeks
10. Individuals with teeth that had deep periodontal pockets (> 5 mm)
11. Individuals who underwent cervical restorative treatment (e.g., composite resin and glass ionomer)

**Figure 1:**
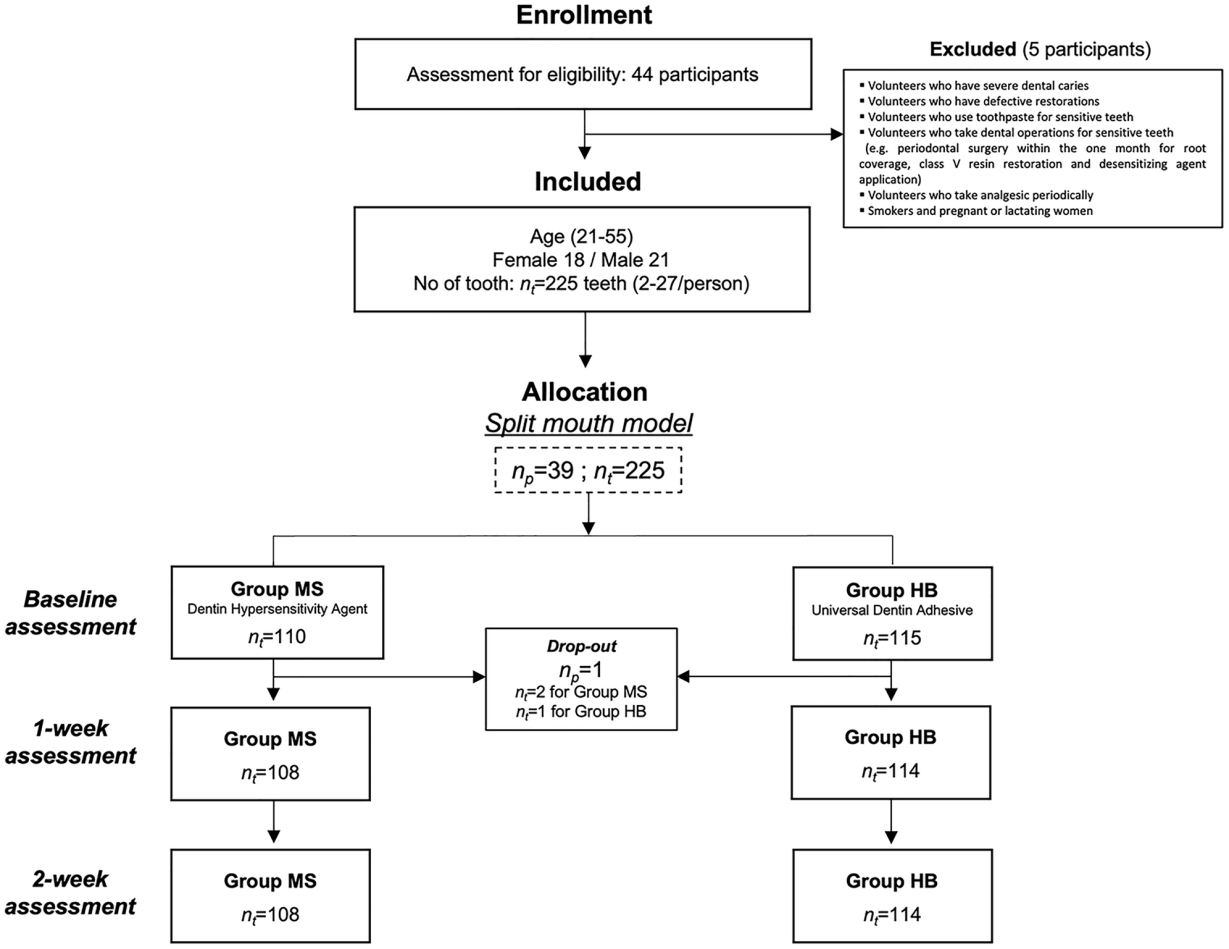
The consolidated standards of reporting trials flow diagram of the clinical trial. Abbreviations: n_p_, number of participants; n_t_, number of teeth; MS, MS Coat ONE; HB, Hi-bond universal.

Fifty-two patients volunteered to participate in this study. Before enrollment in the clinical trial, a survey was conducted to identify DH, and participants who did not meet the criteria were excluded. Consequently, 44 participants (aged 18–65 years) were enrolled. They underwent a primary screening assessment before the study using an air-blast test and were evaluated using the SSS (Fig. 1). Participants who had more than two sensitive teeth with an SSS ≥ 2 in different quadrants (2–4 quadrants in one participant) were included in the study.

Each quadrant in a participant was randomly grouped into (1) Group MS, treated with a conventional desensitizing agent (MS Coat ONE; Sun Medical, Moriyama, Japan); and (2) Group HB, treated with an MBG-containing universal dentin adhesive (Hi-Bond Universal; MEDICLUS, Cheongju, Korea). The compositions of the experimental agents used in this study are listed in Table 1.

**Table 1:** Composition of experimental materials.

Group	Product (Manufacturer)	Composition (or ingredients)	Application methods
MS	MS coat ONE (Sun Medical, Japan)	Poly-styrene sulfonic acid, poly-methyl methacrylate (PMMA), and oxalic acid	Clean the tooth surface with a moistened cotton pledget. Wipe with a dry cotton pledget Dispense 1–3 drops of MS Coat ONE into the plastic dispensing dish Control moisture with cotton rolls. Apply the liquid with a felt applicator for 30 s using a pumping/rubbing motion. Then gently dry the surface with an air syringe for 10 s. If the surface remains hypersensitive, repeat the procedure
HB	Hi-bond universal (MEDICLUS, Korea)	Mesoporous bioactive glass, 10-methacryloyloxydecyl dihydrogen phosphate, ethanol, methacrylate resin monomer, silane coupling agent, camphorquinone, ethyl-4-(dimethylamino)benzoate, and dibutyl hydroxytoluene	Control moisture with cotton rolls Clean the prepared cavity with water Apply 1–2 drops of Hi-Bond with a microbrush and agitate for 20 s. Lightly air-dry to evaporate the solvent. Use a curing light to polymerize the adhesive for 10 s

Five participants were excluded due to severe dental caries, defective restorations, or having undergone dental procedures for DH (e.g., periodontal surgery for root coverage, class V resin filling, and desensitizing agent application) within the past 4 weeks before the study. Smokers and pregnant or lactating women were also excluded (Fig. 1). Thirty-nine participants were finally included in the clinical trial. A summary of the participant’s demographic characteristics is presented in Table 2.

**Table 2:** Baseline characteristics of the included participants.

Participants	Variables		Overall characteristics (n_p_ = 39)	
	Age (years)	Mean (SD)	35.04 (9.86)	
		Median	34.00	
		Min–Max	21–55	
	Sex	Male	21 (53.8%)	
		Female	18 (46.2%)	

Allocated teeth	Variables	Group MS (n_t_ = 110)	Group HB (n_t_ = 115)	*p*-value
Localization of DH teeth	Maxilla	52	62	0.6667
	Mandible	58	53	
Type of DH teeth	Incisors	38	34	> 0.9999
	Premolars	40	51	
	Molars	32	30	
Mean Schiff sensitivity score at screening (SD)		2.20 (0.70)	2.10 (0.63)	0.2608

*DH*, dentin hypersensitivity; *SD*, standard deviation.

### Randomization

The participants had 2–4 quadrants with dentin hypersensitivity with an SSS ≥ 2. A split-mouth design was employed, and each participant’s teeth were eventually treated with both agents. Each quadrant was assigned an alphabetical code (A or B). Randomization was performed using a table of random numbers (one or two; Group MS or HB) that were computer-generated by a researcher blinded to the type of agent used. Each quadrant was treated with an identical agent. The researcher who prepared the randomization code (SO) was not involved in participant screening or examination at any time point. A total of 225 teeth were allocated to the two experimental groups (MS or HB). The Groups MS and HB included 110 and 115 teeth, respectively (Table 2).

### Clinical assessment

Patients were assessed at baseline, after the application of the agents, and at the 1- and 2-week follow-ups. Two authors (HK and DK) conducted the assessments. During each assessment, the tooth surface was exposed to three different stimuli: air, cold, or acid. Each stimulus was applied according to a standardized protocol, with an interval of 5 min, as described by Sowinski et al.^15^. Subjective discomfort associated with DH was assessed using the VAS at every visit. The clinical examiners of the blinded team, participants, and outcome adjudicators were blinded to the allocation of experimental groups throughout the study. Each assessment was conducted at baseline, immediately post-application, and at the 1- and 2-week follow-ups.

1. Air: Air was delivered from a standard dental unit air syringe at approximately 1 cm for 5 s and 3.5–4.0 bar at an operating temperature of 23 °C (± 4 °C), directed at the exposed cervical surface of the hypersensitive tooth. The SSS was used to assess the participants’ response to this stimulus, as follows^27^
  0 = The participant did not respond to the air stimulus
  1 = The participant responded to the air stimulus but did not request its discontinuation
  2 = The participant responded to the air stimulus and requested its discontinuation or moved away from it
  3 = The participant responded to the air stimulus, considered it to be painful, and requested discontinuation.
2. Cold: Thermal stimuli were applied to the teeth for 1 s using a microbrush (fine size, 1.5 mm in diameter; Microbrush International, Wisconsin, USA) sprayed immediately with ethyl chloride ice spray (Walter Ritter, Hamburg, Germany). The SSS was used to assess the participants’ responses to cold stimuli, following the standard suggested by Schiff et al.^27^.
3. Acid: A 20% lemon solution (Fior di Limone; Ital Lemon SPA, Lodi, Italy) was used as an acidic stimulus to assess DH. This solution was applied to the teeth for 3 s using a microbrush at room temperature and scored using the SSS ^27^.
4. VAS: At every visit, the subjective DH of the entire dentition of participants was rated based on VAS, with 0 denoting “none” and 10 denoting “extremely severe” DH at pre-application, immediately post-application, and at the 1- and 2-week follow-ups. VAS was used to measure the general discomfort caused by DH. VAS scores were recorded as case records.

After the clinical evaluation was performed by the blinded team (HK and DK) at baseline, two operators (JJ and JK) from the unblinded team applied the two agents according to a random code, as per the manufacturer’s instructions (Table 1). Before the application of both agents, screened and randomized teeth were cleaned with pumice. After agent application, the clinical evaluation was performed by the blinded team immediately post-application and at the 1- and 2-week follow-ups.

### Safety report

Each participant was interviewed about adverse events at each visit. The soft and hard tissues of the oral cavity were visually examined at every visit to assess product safety. We planned that any adverse events that occurred during the study would be reported and recorded immediately.

### Statistical analyses

The primary outcome of this study was the difference in the SSS for air, cold, and acidic stimuli, and the secondary outcome was the difference in VAS scores. In test groups, the mean SSS for each stimulus modality was calculated for patients and teeth during each assessment period. Descriptive statistics were calculated for each measurement and experimental group. Differences in the SSS between the follow-ups and experimental groups were evaluated using a linear mixed-effects model. The level of significance was set at α = 0.05. Statistical computations were performed using SPSS (25.0.0, IMB Corp., Armonk, NY, USA) and GraphPad Prism (version 10.0, Boston, MA, USA).

## Results

### Baseline summary

Of the 44 participants enrolled in the study, five were excluded. Finally, 39 participants were included, and the teeth with DH were allocated using a split-mouth model. Among them, one participant was excluded from the study due to loss of contact, leaving 38 participants who completed the study. A total of 110 teeth were allocated to Group MS and 115 to Group HB. Data from 108 teeth in Group MS and 114 teeth in Group HB were acquired. The study population had a mean age of 35.04 years (range, 21–55 years). Patient demographics are presented in Table 2. The demographic features (sex distribution, mean age, tooth position, and the mean SSS to air blast) at baseline were comparable between the control and test groups (*p* > 0.05).

### Efficacy assessment

**Figure 2:**
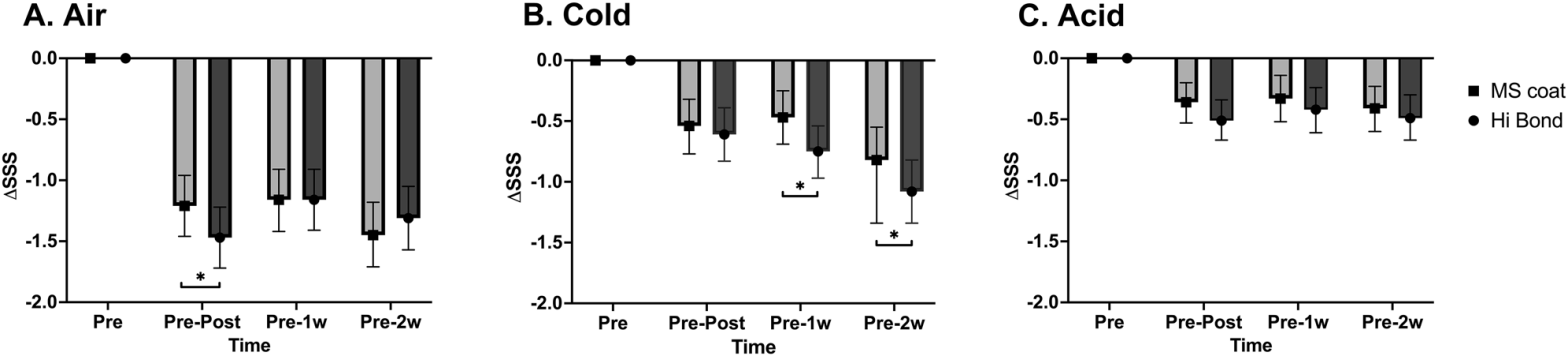
Schiff sensitivity score (SSS) changes (ΔSSS, least squares means and 95% confidence interval). The SSS was measured at baseline (pre-application), immediately after applying the agent, and at 1- and 2-week follow-ups following (1) air, (2) cold, (3) and acid stimuli. Light gray bars present the data of Group MS, while dark gray bars present the data of Group HB. *Below the bar denotes statistically significant difference between groups (*p* < 0.05).

**Figure 3:**
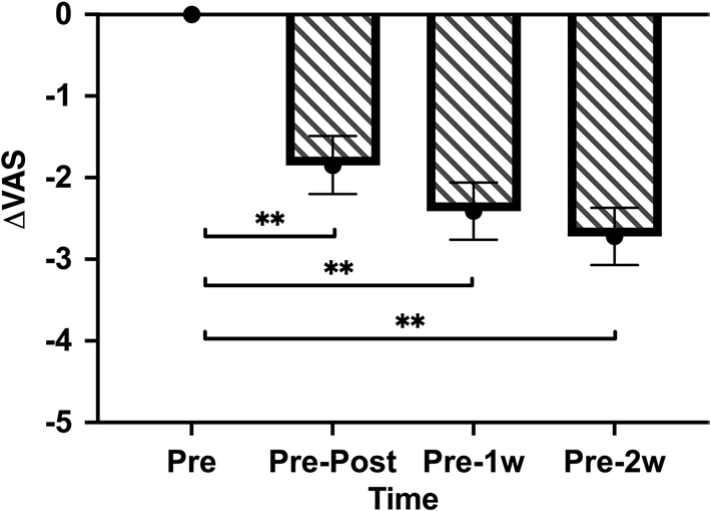
Visual analog scale (VAS) changes (ΔVAS, least squares means and 95% confidence interval) demonstrating general discomfort during the clinical trial. **Below the bar denotes statistically significant difference between the assessment periods (*p* < 0.01).

This study was sufficiently powered (1 − β ≈ 1.00) to demonstrate the clinical equivalence or superiority of Group HB to Group MS. A mixed-effects model was used for statistical analysis with adjustments for age, sex, and tooth position (maxilla or mandible; incisor, premolar, and molar). Figure 2 demonstrates the SSS changes (ΔSSS and 95% confidence interval) calculated immediately post-application and at 1- and 2-week follow-ups compared with those at baseline (pre-application) for (1) air, (2) cold, and (3) acidic stimuli. After the application of each agent (immediately post-application and at 1- and 2-week follow-ups), the SSS significantly decreased in both groups for all stimuli. Participants reported decreased discomfort associated with DH at the 2-week follow-up (Fig. 3).

1. Air.
  Both groups exhibited rapid relief from perceptual DH immediately after the application of desensitizing agents; however, the DH relief did not intensify over time (Table 3). In the intergroup comparison, the reduction in the SSS post-application was significantly greater in Group HB than in Group MS (*p* = 0.0178) (Fig. 2a). Although both Groups MS and HB demonstrated a consistent decrease in the SSS, no significant difference was observed in the reduction in air sensitivity scores between Groups MS and HB at the 1- and 2-week follow-ups (*p* = 0.9723; *p* = 0.1134).

**Table 3:** Statistics of the Schiff sensitivity score and post-hoc analysis of the mixed-effect model for repeated data (mixed-effect model; LS mean and 95% confidence interval).

Schiff sensitivity score	Adjusted (LS) mean (95% CI)		Bonferroni post-hoc (*p*-value)			
	Group MS	Group HB	Group	Time		
			MS versus HB	Pre versus post	Pre versus 1 w	Pre versus 2 w
Air						
Pre	2.18 (2.01, 2.34)	2.13 (1.97, 2.30)	–			
Post	0.96 (0.71, 1.22)	0.67 (0.41, 0.92)	**0.0178**	0.0612	0.7144	0.1772
1 w	1.00 (0.74, 1.25)	0.96 (0.71, 1.22)	0.9723			
2 w	0.69 (0.44, 0.94)	0.81 (0.56, 1.06)	0.1134			
Cold						
Pre	1.83 (1.56, 2.11)	1.90 (1.63, 2.17)	–			
Post	1.28 (1.02, 1.55)	1.27 (1.01, 1.53)	0.6382	0.7450	0.1401	0.1978
1 w	1.35 (1.09, 1.62)	1.15 (0.89, 1.41)	**0.0231**			
2 w	1.01 (0.75, 1.26)	0.81 (0.56, 1.06)	**0.0377**			
Acid						
Pre	0.62 (0.41, 0.84)	0.71 (0.50, 0.92)	–	0.2552	0.3857	0.4623
Post	0.28 (0.15, 0.39)	0.22 (0.10, 0.34)	0.1450			
1 w	0.31 (0.17, 0.45)	0.28 (0.14, 0.43)	0.3596			
2 w	0.24 (0.10, 0.39)	0.23 (0.08, 0.37)	0.5365			
VAS (SD)						
Pre	4.98 (1.53)			** < 0.0001**	** < 0.0001**	** < 0.0001**
Post	3.15 (1.42)					
1 w	2.59 (1.41)					
2 w	2.28 (1.41)					

*LS,* least squares; *CI*, confidence interval; *VAS*, visual analog scale; *SD*, standard deviation; Pre, baseline; Post, post-application; 1w, 1-week follow-up; 2w, 2-week follow-up. Bold values indicate the *p*-value data with *p* < 0.05.
2. Cold.
  Both groups demonstrated a reduction in the SSS in response to cold stimuli after the application of the agents (immediately post-application and at the 1- and 2-week follow-ups). However, the reduction in the SSS was gradual according to the follow-up period and was cumulative. Immediately after applying agents, Groups MS and HB displayed no significant difference in the reduction in the SSS (Fig. 2b). At the 1- and 2-week follow-ups, a significantly higher reduction in the SSS was observed in Group HB than in Group MS (1-week follow-up, *p* = 0.0231; 2-week follow-up, *p* = 0.0377).
3. Acid.
  The two groups displayed alleviating effects of acidic sensitivity at three-time points (immediately post-application and at the 1- and 2-week follow-ups). However, no significant intergroup differences were observed in the extent of SSS reduction at any follow-up point. (Fig. 2c).
4. VAS.
  Subjective discomfort due to DH was assessed using the VAS, as this was a split-mouth clinical trial. Regarding DH, no group comparisons were conducted. Participants demonstrated a gradual decrease in the SSS immediately post-application and at the 1- and 2-week follow-ups (Fig. 3). The mean VAS scores and standard deviations at each follow-up visit are presented in Table 3. The reduction in DH, presented by the participants, was evident during the follow-up period (immediately post-application and at the 1- and 2-week follow-ups) (*p* < 0.0001) (Table 3).

### Safety reports

Over the 2-week follow-up period, no adverse incidents were reported.

## Discussion

The diagnosis and management of DH continue to pose significant challenges in dental practice^2^. DH diagnosis can be complicated due to the subjective nature of patient reports and the multifactorial etiology of the condition^28,29^. The varied causes of DH also complicate treatment approaches. Patient perceptions of pain in response to various stimuli, such as air and cold, can vary significantly. Although the effects of various DH treatments have been proven, the precise mechanism for suppressing sensitivity to different stimuli has not yet been fully elucidated. In the present study, the clinical efficacy of the two interventions in response to various stimuli was identified by administering two different desensitizing agents to the same participant. The desensitization pattern varied depending on the stimulus type.

This study observed an immediate and significant reduction in SSS in both groups following exposure to air stimuli. This finding is consistent with those of the previous studies demonstrating the rapid action of desensitizing agents in alleviating hypersensitivity triggered by air stimuli^30^. Notably, the immediate reduction in DH was significantly greater in Group HB than in Group MS. According to the manufacturer’s instructions, MS Coat ONE was not light-cured after application and chemically reacted with the tooth substrate, although the chemical reaction did not occur immediately. Hi-Bond Universal was light-cured and promptly formed a stable adhesive layer on the hypersensitive dentin surface. However, this effect plateaued over the follow-up period, indicating that DH relief did not intensify further beyond the initial reduction. Thus, the first null hypothesis was partially rejected.

The study identified a progressive reduction in the SSS for cold stimuli over the follow-up period. This result is consistent with the gradual and cumulative effects of treatment interventions for the management of cold-induced DH. Although both groups exhibited a similar trend, Group HB displayed a significantly greater reduction in SSS than that observed with Group MS at the 1- and 2-week follow-ups. This indicates that the MBG contained in Hi-Bond Universal may occlude the dentin surface owing to its remineralization potential. Our in-vitro study reported that the reduction in the dentinal fluid flow rate increased with time when bioactive glass containing experimental desensitizers was employed^31^. Remarkably, the reduction in the DH increased over time.

In contrast to the discernible responses to air and cold stimuli, this study identified no significant differences between the two groups in terms of the reduction in the SSS for acidic stimuli. This finding indicates a similar efficacy of both interventions in the management of acid-induced DH. The lack of a differential response may reflect shared mechanisms through which desensitizing agents and universal adhesives mitigate acidic hypersensitivity.

A subjective assessment using the VAS revealed that participants reported a gradual decrease in overall discomfort due to DH during the follow-up period. Thus, the second null hypothesis was rejected. This aligns with the objective findings of reduced SSS and underlines the clinical relevance of interventions to enhance the well-being of patients. This study’s focus on the oral discomfort of patients provides an overall understanding of treatment efficacy, as patients experience hypersensitivity regardless of the split inspection areas of the mouth.

Both agents in this study exhibited desensitizing effects by blocking the dentinal tubules, although their active ingredients differed. The oxalates contained in MS Coat ONE occlude the dentinal tubules by reacting with calcium ions, causing a precipitation reaction that leads to the formation of insoluble calcium oxalate crystals^32^. MBG in Hi-Bond Universal, such as sodium, calcium phosphosilicate, or calcium phosphate, promotes the formation of apatite hydroxycarbonate, similar to hydroxyapatite, on the dentin surface, thereby blocking the dentinal tubules^4^. This study focused on the efficacy of two different in-office desensitizers for managing DH. An in vitro study conducted by Bakry et al. demonstrated that phosphoric acid-treated bioglass exhibited superior dentinal tubule occlusion, compared to that observed with oxalate-containing MS Coat ONE^33^. The effect reportedly persists even after artificial brushing abrasion. Bakry et al. revealed that the thin resin copolymer in MS Coat ONE was quickly removed by tooth brushing^33^. As the resin copolymer in MS Coat ONE assists in the reaction between oxalate and calcium, removal of the copolymer might hinder effective tubule occlusion by oxalate. In contrast, the polymerized dentin adhesives in Hi-Bond Universal may act as a barrier. This enables MBG to actively promote mineralization at the entrance of the dentinal tubules at the adhesive–dentin interface. This may help ensure completion of the bioactive stage (2–10 h), which Hench proposed as the necessary time for the remineralization reaction^34^. Owing to the porous structure of MBG, the increased surface area and enhanced reactivity lead to rapid ion release and swift formation of hydroxycarbonate apatite, which can precipitate and block the dentinal tubules effectively. The size and interconnected nature of the pores in the MBG enhance this interaction with the biological environment, speeding the occlusion process and providing a reliable tubule seal.

These results suggest distinct response patterns to the interventions, offering valuable insights into the chronological dynamics of DH relief and its subjective perception. The observed variations could be attributed to differences in patients’ perception thresholds and adaptation to specific stimuli, which might have introduced some level of bias into the results. In this study, we report differential responses to air and cold stimuli. This could be attributed to the differences in the mechanisms of action, wherein air stimuli predominantly affect open dentinal tubules, whereas cold stimuli may involve nerve responses through temperature changes and mechanoreceptor excitation by fluid movement^35^. Studies have proposed that dentinal fluid flow may not be the only mechanism involved in the thermal sensation in human teeth^36^. If the tooth expands or contracts due to thermal stimulation, it may not only exert a physical effect on mechanical fluid movement but also directly distort odontoblastic processes inside the tubules, which in turn induces nerve stimulation^35^. Byers et al. reported that hydrodynamic pressure and intense cooling can activate other types of intradental nerve fibers^37^. We believe that this aligns with the findings of the present study. Partial occlusion at the entrance of the dentinal tubules from DH treatment prevents fluid movement in response to air stimuli, resulting in immediate desensitization. If cold stimuli cause fluid movement in dentinal tubules and directly stimulate nerve endings near the dentinoenamel junction by thermal stimuli, occlusion not only at the tubule entrance but also deep within the tubules should provide sufficient physical resistance to decrease temperature transmission. This may explain the gradual alleviation of DH by cold stimuli.

The results of this study have clinical implications in the management of DH. Immediate relief following air stimuli, sustained reduction in cold hypersensitivity, and comparable efficacy in managing acidic sensitivity highlight the feasibility of the use of both MS Coat ONE and Hi-Bond Universal for DH relief. This study suggests that in a clinical environment, using a universal dentin adhesive containing MBG could be an effective option for DH relief when a dedicated desensitizing agent is unavailable, potentially offering superior performance in some cases.

Acknowledging the limitations of this study is important, including the relatively short follow-up period and restricted focus on specific stimuli. Future research should explore the long-term efficacy and potential relapse of hypersensitivity symptoms beyond the 2-week window. In addition, investigations into the mechanisms underlying differential response patterns may provide further insights into the effectiveness of interventions.

In conclusion, this study provides valuable insights into the temporal dynamics of DH relief following treatment with a dedicated desensitizing agent containing oxalate/resin copolymer and a universal dentin adhesive containing MBG. Both agents offer immediate relief in air-induced DH without significant change in effectiveness over time. Notably, an MBG-incorporated universal adhesive demonstrated significantly greater relief from air sensitivity than that with the dedicated desensitizing agent. Regarding cold stimuli, both treatments exhibited a progressive and sustained desensitizing effect, with no significant difference between the groups. This research suggests the efficacy of an MBG-incorporated universal dentin adhesive in alleviating DH, demonstrating that it is comparable to or better than that of a dedicated desensitizing treatment. Furthermore, the alleviating effects of these agents differ with various stimuli, suggesting that clinicians can anticipate the degree and pattern of relief when using these modalities for DH management.

## Data Availability

The datasets generated and analyzed during this study are not publicly available due to ethical restrictions of clinical trials; however, they are available from the corresponding authors (J.-H. Jang or D.-S. Kim) on reasonable request.
